# Comparative Ion Channel Transcriptomes of NK1R and Somatostatin Neurons in the preBötzinger Complex of the Ventrolateral Medulla

**DOI:** 10.21203/rs.3.rs-9752257/v1

**Published:** 2026-07-02

**Authors:** Hemalatha Bhagavan, Aguan D. Wei, Luiz M. Oliveira, Jan-Marino Ramirez

**Affiliations:** Seattle Children’s Research Institute; Seattle Children’s Research Institute; Seattle Children’s Research Institute; Seattle Children’s Research Institute

**Keywords:** preBötzinger, single-cell transcriptomics, ion channels, neurokinin-1 receptor, tachykinin receptor 1

## Abstract

The preBötzinger Complex is among the few neural circuits where selective elimination of defined neuronal subpopulation is sufficient to destabilize a core autonomic function and can fatally impair breathing. Within this circuitry, neurons expressing the neurokinin-1 receptor (*Tacr1/NK1R*) and somatostatin (*Sst*) are critical subpopulations; *Tacr1*^+^ neurons respond to the neuropeptide substance P and are necessary for maintaining inspiratory rhythms, and ablation of *Sst*^+^ neurons results in apneas. To further dissect and analyze the specific roles for *Tacr1*^+^ and *Sst*^+^ cell types, we conducted a comprehensive transcriptomic analysis using single-nucleus RNA sequencing of enriched preBötC from neonatal C57BL/6J mice. Because respiratory rhythmogenesis is an inherently electrophysiological process, we focused on the ion channel transcriptomes of Tacr1 + and Sst+ populations to resolve the molecular underpinnings of their distinct contributions to breathing. A balanced Random Forest classifier distinguished Tacr1^+^ from Sst^+^ neurons with higher accuracy, indicating a distinct and relatively homogeneous ion channel identity in Tacr1^+^ neurons. Differential expression analyses identified coordinated upregulation of *Trpc5*, *Kcnc2* and *Cacna2d2* genes in Tacr1^+^ neurons. Tacr1^+^ neurons further exhibited elevated expression of the NALCN channelosome subunits and selective enrichment genes of *Htr2c* and *Adra1a* neuromodulatory receptor genes. Together, these findings define a molecularly distinct ion channel composition of Tacr1^+^ neurons and imply convergent substance P linked mechanisms: TRPC5-mediated I_CAN_ and NALCN-mediated sodium leak conductance supporting rhythmic inspiratory activity, providing a molecular framework for targeted interrogation of respiratory circuit function.

## Introduction

Breathing is vital and generated by neuronal networks within the lower brainstem. Investigations into identifying the neuronal network(s) underlying the generation of the respiratory rhythm is an area of active ongoing research, and a better understanding of how breathing is regulated at the level of specific identifiable neuronal types would provide a crucial step for devising targeted prevention therapies in case of disorders associated with breathing disturbances or sudden death associated with the failure to breath [1]. The preBötzinger Complex (preBötC) within the Ventral Respiratory Column (VRC) is the core region responsible for generating the inspiratory rhythm [2–6]. Ablation of this region leads to cessation of breathing [7–10]. The inspiratory rhythm activity generated by the preBötC is critically dependent upon the embryonic expression of the homeobox transcription factor *Dbx1* gene in V0 precursor cells of the developing medulla between E10.5 to 11.5 [11]. In the absence of *Dbx1* expression, the preBötC fails to generate rhythmic bursting activity, disrupting the core neural circuits required for respiration in the developing embryo. This also leads to a complete loss of somatostatin (*Sst*) and the neurokinin 1 receptor (NK1R/*Tacr1*) expressing neurons in the preBötC [11–20]. Conversely silencing this Sst^+^ population led to apnea in adult rats [21], and specific and near complete bilateral destruction of preBötC NK1R neurons results in ataxic breathing [10]. These findings emphasize the essential role of Sst^+^ and Tacr1^+^ neuronal population in maintaining respiratory rhythm.

To gain insights into the specific role of Sst^+^ and Tacr1^+^ expressing neuronal population in inspiratory rhythm generation, researchers have integrated pharmacological, genetic, and viral tracing approaches with electrophysiology [21–31]. Here, we further characterize these populations by profiling ion channel gene expression at the single-nucleus level. To this end, we performed single-nucleus RNA sequencing (snRNA-seq) on micro-dissected preBötC-enriched brainstem tissue from 7 neonatal C57BL/6J mice (P5–P10), yielding a dataset from which we identified 465 Sst^+^ and Tacr1^+^ nuclei for downstream analysis. Our findings reveal distinct ion channel transcriptomic signatures for these two populations, supporting the notion that they serve specialized roles in respiratory rhythmogenesis.

We examined whether Sst^+^ and Tacr1^+^ neuronal populations could be distinguished based on their ion channel transcriptomes, despite sharing a common *Dbx1* progenitor lineage. Using a Random Forest classifier, we assessed classification performance and found that Tacr1^+^ neurons were identified with significantly greater accuracy than Sst^+^ neurons. We then performed differential expression (DE) analysis to highlight key transcriptomic features and found enrichment of ion channel genes in Tacr1^+^ neurons, including potassium channels such as *Kcnc2*, the voltage-gated calcium channel auxiliary subunit *Cacna2d2*, and the TRP channel *Trpc5*. We next evaluated components of the NALCN sodium leak channel complex and observed differences consistent with altered baseline excitability, alongside distinct neuromodulatory GPCR profiles enriched in the Tacr1^+^ population. Finally, we integrated these findings to demonstrate that Tacr1^+^ neurons exhibited clear transcriptomic specializations within the preBötC/VRC [27], suggesting molecular mechanisms that may underlie their role in respiratory rhythm generation and modulation.

## Materials and Methods

### Animals

Neonatal (P5–P10) C57BL6/J mice bred at Seattle Children’s Research Institute, were used for sample collection in compliance with the guidelines of the Seattle Children’s Research Institute Animal Care and Use Committee and the National Institutes of Health. All animals had free access to water and food and were housed in a temperature-controlled (22 ± 1°C) facility with a 12:12 h light/dark cycle.

### Tissue Processing

Animals were deeply anesthetized with 3.0% isoflurane and decapitated, after which isolated brainstems were immediately placed in ice-cold artificial cerebrospinal fluid (aCSF with an osmolarity between 305–312 mOsm, containing (in mM): 118 NaCl, 3.0 KCl, 25 NaHCO_3_, 1 MgCl_2_, 1.5 CaCl_2_, and 30 d-glucose equilibrated with carbogen (95% O_2_, 5% CO_2_) at pH 7.4 ± 0.05. Brainstems were affixed on 5% agar blocks with cyanoacrylate glue and allocated on a vibratome (VT1000S, Leica Microsystems, Waukegan, IL) with the rostroventral edge facing towards the blade for serial transverse sectioning along the rostralcaudal axis. Serial sectioning continued until the caudal edge of the facial nucleus was reached, after which a 400 micron slice was taken to expose the rostral edge of the preBötC [32–34]. A single 600 μm slice was then made, containing the entire preBötC. preBötC regions located immediately ventral to the Nucleus Ambiguus (NA) were punched out and collected bilaterally (n = 7). All tissue collections were performed in oxygenated aCSF prechilled on ice and stored at −80° until further processing. Proper care and ethical guidelines were followed for all animal handling and sample collection, under IACUC protocol #0058 approved by Seattle Children’s Research Institute (SCRI).

### Single Nuclei Preparation

Single-nucleus suspensions were obtained as previously reported [35]. Briefly, we followed the 10X Genomics protocol (CG000366) with key modifications. First, Tween-20 was excluded from the working wash buffer. Second, the lysis buffer was supplemented with Nonidet-P40 Substitute (Sigma, 74385) at a final concentration of 0.5% and Tween-20 at 0.1%, per the manufacturer’s recommendation. Frozen tissues were mechanically dissociated in sterile 1.5 mL microcentrifuge tubes using a 1.5 mL pellet pestle in 1X lysis buffer and incubated for 10 minutes to release free nuclei. The suspension was then centrifuged at 500 rcf for 5 minutes at 4°C. The resulting nuclear pellet was washed, resuspended in a wash buffer, and passed through a 70 μm filter (pluriStrainer Mini 43–10070–40). These modifications facilitated the successful isolation of nuclei for downstream analysis. All steps were conducted on ice.

### Single Nuclei RNA sequencing

We used 7 brainstem samples of C57BL6/J mice between P5 to P10. They were micro-dissected and pooled to enrich for the preBötC region. Pooled nuclei were processed in two technical replicates. Barcoded cDNA libraries were prepared using the Chromium Single Cell 3′ Library & Gel Bead Kit v2 (10xGenomics). Sequencing was performed on an Illumina NovaSeq 6000 (S1 flow cell) to a total depth of 190 million reads (Northwest Genomics Center, University of Washington, Seattle, WA). Raw sequencing reads were processed to count matrixes with Cell Ranger (version 2.2.1) with default parameters, using the mm10 mouse genome as reference. All statistical analyses were performed under R (version 4.4.1).

### Quality Control, Preprocessing, Normalization, Clustering and Annotation

Expression matrix files were analyzed using the Seurat v5.0 package [36]. To eliminate ambient RNA contamination SoupX [37] was applied. We excluded nuclei by the criteria of nFeature_RNA > 800, nCount_RNA > 1000, and percent.mt < 15%. Cell cycle scoring was implemented to check the effects of cell cycle heterogeneity. The data were normalized using the sctransform (SCT) method with glmGamPoi, incorporating the proportion of mitochondrial and cell cycle genes as a variable to regress out [38, 39]. Principal components were computed, and key dimensions were selected based on association with genes of interest (*Slc17a6, Slc17a7, Chat, Gad1, Gad2, Slc6a5, Slc32a1, Syt1, Mag, Mog, Aldh1l1*) within the loading matrix. The top dimensions corresponding to genes of interest were then used to construct the nearest neighbor graphs, while adjusting for correlations between average expression and dispersion. Uniform Manifold Approximation and Projection (UMAP) was performed using the key dimensions from principal components (PCs) with a modularity optimization Louvain algorithm. Clustering was optimized using Seurat with a default resolution parameter yielding a dataset of 7872 nuclei of preBötC. SingleR (Single-cell Recognition) was used in annotating clusters by assigning cell type labels to individual cells based on their transcriptomic profiles by comparing them to built-in reference gene expression data of a mouse database. Using correlation and statistical methods, SingleR determines the most likely cell identity for each cell, providing accurate and automated annotations. We subsetted neurons of preBötC. From the neuronal cluster of 4751 nuclei, Tacr1^+^ and Sst^+^ neurons were isolated by subsetting cells with normalized gene expression of *Tacr1* and *Sst* genes greater than 0.5. Cell type designations follow the gene symbol (no italics, first letter capitalized) with a superscript +, indicating a population defined by expression of that marker gene (e.g., Tacr1^+^, Sst^+^).

### Ion Channal and Receptor Analysis

For all ion channel analyses described in this study, we curated a reference list of 229 ion channel genes, of which 192 were detected in the dataset across the Sst^+^ and Tacr1^+^ populations. Additionally, 62 receptor genes were curated, all of which were detected in the dataset. The complete annotated gene list, including family classifications, is provided in Table S1.

### Random Forest (RF) Analysis

For the random forest analysis, two sequential filtering steps applied to the original 192 detected ion channel genes which lead to 118 ion channel genes. First, we retained ion channels expressed in at least 10% of all cells combined (pct_total > 10%), thereby removing very lowly expressed or near-absent channels from the dataset. Second, we applied a minimum detection threshold of > 3.2% in Sst^+^ cells. This prevents bias towards falsely improving the classification performance towards larger group, Tacr1^+^ cells, n = 313. To address the unequal group sizes (Sst^+^: 152 cells; Tacr1^+^: 313 cells) and reduce sampling bias, we used a balanced iterative resampling approach. We ran 200 iterations that randomly drew an equal number of cells from each population (matching the smaller group, that is the Sst^+^ population), trained a Random Forest model on the balanced subset using 500 trees, and recorded model performance with cell type as the outcome variable and the 118 filtered ion channel genes as features. Random Forest models [40, 41] were implemented using the randomForest package in R. At each node, the best split was selected using Gini impurity as the splitting criterion, with the number of features randomly sampled at each split set to the default value of √p, where p is the total number of features. Bootstrap samples for each tree were drawn with replacement from the balanced subset, with the remaining out-of-bag samples used as an internal test set for that tree. Model performance was evaluated using OOB error estimates, and per-class accuracy was extracted from the OOB confusion matrix. Feature importance was assessed using mean decrease in accuracy, aggregated across all 200 iterations. To further evaluate discriminability, ROC curves were computed for each iteration using the pROC package, with predicted class probabilities for Tacr1^+^ cells as the classifier score. Mean AUC and standard deviation were calculated across iterations as a summary measure of overall classification performance. To validate that OOB accuracy estimates generalized beyond the balanced training subset, a stratified held-out evaluation was additionally performed. In each iteration, the 161 Tacr1^+^ cells not selected for training were withheld entirely from forest construction and evaluated using out-of-sample prediction via the trained model. Held-out Tacr1^+^ accuracy was computed as the proportion of correctly classified cells and compared against the OOB accuracy of the training subset across all 200 iterations. Unlike OOB accuracy, which aggregates bootstrap votes across 500 trees for each training cell and thereby dampens iteration-to-iteration variance, held-out accuracy was computed as a single proportion across 161 withheld cells whose composition varied across iterations. This difference in estimator structure means that sampling variance across Tacr1^+^ cell draws is more directly visible in the held-out distribution, which is expected to be wider than the OOB distribution despite comparable means. All results, including accuracy distributions, confusion matrices, ROC curves, and feature importance scores were derived from a single forest per iteration to ensure internal consistency.

### Differential Expression (DE) Analysis

Seurat’s “FindMarkers” function was used to identify DE genes between population comparison. The Wilcoxon rank-sum test with Benjamini–Hochberg correction was applied to obtain adjusted P values. Genes with an FDR-adjusted P value of < 0.05 and an absolute log2-fold change of > 0.5 were considered differentially expressed. To complement the analysis, Fisher’s exact tests were performed on binary detection rates for the candidate ion channel genes identified from the differential expression results. For each gene, detected cell counts per population were derived from the proportion of expressing cells reported by FindMarkers, and contingency tables were constructed for each gene and corrected with Benjamini–Hochberg. Effect sizes were calculated as Cohen’s *d* using the difference in mean normalized expression between Tacr1^+^ and Sst^+^ neurons divided by the pooled standard deviation.

### Pseudobulk Analysis

To evaluate the directional consistency of ion channel gene enrichment across analytical approaches, an exploratory pseudobulk analysis was performed. Raw counts for the 118 filtered ion channel genes were aggregated by cell type and technical replicate using the “AggregateExpression” function in Seurat, yielding four pseudobulk samples (Tacr1^+^ and Sst^+^ across two technical replicates). Aggregated counts were normalized to counts per million (CPM) to account for differences in the number of cell contributing to each pseudobulk sample, followed by log2 transformation (log2(CPM + 1)). For each gene, log2 fold change was calculated as the difference in mean log2CPM between Tacr1^+^ and Sst^+^ groups across replicates. No formal statistical testing was performed, as the dataset comprised two technical replicates rather than independent biological replicates, used solely to assess the directionality of expression differences for genes identified as discriminatory by RF classification and to confirm consistency with Fisher’s exact enrichment results.

#### Module scoring of NALCN channelosome gene signature.

Module scores for the NALCN channelosome were calculated using the “AddModuleScore” function in Seurat [42], which computes the average expression of a defined gene set relative to randomly sampled background genes of similar expression levels. The comprised transcripts encoding the four NALCN channelosome subunits: *Nalcn* (pore-forming), *Unc79*, *Unc80*, and *Fam155a* (auxiliary subunits). For the plotting values below 5th and above the 95th percentile was cut to reduce the influence of outliers on the color scale.

### Histology

Adult mice (n = 3) were deeply anesthetized with 4% isoflurane delivered in 96% oxygen. Transcardial perfusion was performed via the ascending aorta with 20 ml of phosphate-buffered saline (PBS; pH 7.4), followed by 20 ml of 4% paraformaldehyde in 0.1 M phosphate buffer (PB; pH 7.4; Electron Microscopy Sciences, Fort Washington, PA). Brains were carefully extracted and post-fixed in the same fixative at 4°C for 4 hours, then dehydrated in 20% sucrose for 8 hours. Coronal sections (25 μm thick) were obtained using a cryostat and preserved at −20°C in cryoprotectant solution (20% glycerol and 30% ethylene glycol in 50% PB 0.1 M, pH 7.4). The slices were rinsed in PBS, mounted sequentially in rostrocaudal order, air-dried, and kept in −80°C.

*In situ* hybridization was performed using the RNAscope Multiplex Fluorescent Reagent Kit (Advanced Cell Diagnostics). Brain sections were first defrosted in room air, fixed in PFA 4% for 15 minutes and dehydrated sequentially in crescent alcohol concentrations (50%, 70% and 100%, 5 minutes each). The slides were air-dried, and a hydrophobic barrier was drawn around the sections. The slides were then incubated in 0.1M PBS for 5 minutes, followed by treatment with RNAscope Hydrogen Peroxide (#322381) for 10 minutes at room temperature. After three 2-minute PBS washes, sections were incubated with Protease IV (#322381) for 30 minutes at room temperature and then rinsed again in PBS.

Tissue was subsequently hybridized with *Trpc5* (#476241) and *Tacr1* (#428781) probes (Purchased from Advanced Cells Diagnostics, ACD), for 2 hours at 40°C, followed by a brief PBS wash. Signal amplification was carried out using RNAscope Multiplex Detection Reagents (#323110), including sequential incubations with FL v2 Amp 1 and Amp2 for 30 minutes at 40°C, with 5-minute PBS washes between steps. Sections were then incubated with RNAscope Multiplex FL for 30 minutes at 40°C. For HRP-C1 and HRP-C2 signal development, tissues were incubated with the corresponding RNAscope Multiplex FL v2 HRP reagents for 15 minutes at 40°C, followed by PBS washes. Signal detection was achieved using TSA Plus fluorescein (TS-0002000) or Cyanine 3 amplification reagent (FP1170; 1:800) for 30 minutes at 40°C. Finally, sections were treated with RNAscope Multiplex FL v2 HRP blocker for 15 minutes at 40°C, rinsed, mounted with Fluoromount (Thermo Fisher), and cover slipped.

### Cell Counting, Imaging, and Data Analysis

All sections were digitized using a VS120-S6-W Virtual Slide Scanner (Olympus). To minimize bias, imaging and quantification were conducted by a single experimenter blinded to experimental conditions. Cell counts were performed using ImageJ (version 1.41; National Institutes of Health, Bethesda, MD), and schematic illustrations were generated using Canvas software (ACD Systems, Victoria, Canada, version 9.0).

A one-in-two series of 25-μm sections was analyzed per animal, resulting in 50 μm spacing between sampled sections. Data are presented as mean ± SEM. Anatomical alignment for the VRC was performed using a standardized reference section, in accordance with prior work from our laboratory and the Paxinos and Franklin mouse atlas [43].

## Results

### Transcriptomic Analysis of the Tacr1 ^+^ and Sst^+^ neuronal population

To define the molecular architecture of Sst^+^ and Tacr1^+^ neurons at single-cell resolution, we performed snRNA-seq on brainstem tissue enriched for the preBötC region. Tissue from seven C57BL/6J mice (P5–P10) was micro-dissected and pooled for sequencing. Nuclei were isolated using a modified 10X Genomics protocol, and libraries were prepared using the Chromium Single Cell 3′ Kit v3.1. After quality control and filtering (see Methods, Fig. S1a), Tacr1^+^ and Sst^+^ neurons were identified by subsetting nuclei expressing *Tacr1* or *Sst* genes (normalized expression > 0.5), these populations are referred to hereafter as Tacr1^+^ and Sst^+^ neurons, respectively. It is important to note that Tacr1^+^ and Sst^+^ neurons constitute relatively small subsets within the broader neuronal landscape of the enriched preBötC comprising 8.34% (n = 396) and 3.26% (n = 155) of total neurons (n = 4,751), respectively, with only a small fraction (0.32%; n = 15) co-expressing both Tacr1 and Sst (Fig. S1d). This proportional representation is broadly consistent with prior anatomical studies identifying NK1R expressing neurons as a specialized minority population within the preBötC, estimated to comprise approximately 10% of preBötC neurons [27] indicating that the current enrichment strategy preserves physiologically relevant representation of Tacr1^+^ and Sst^+^ neuronal populations.

**Figure 1:**
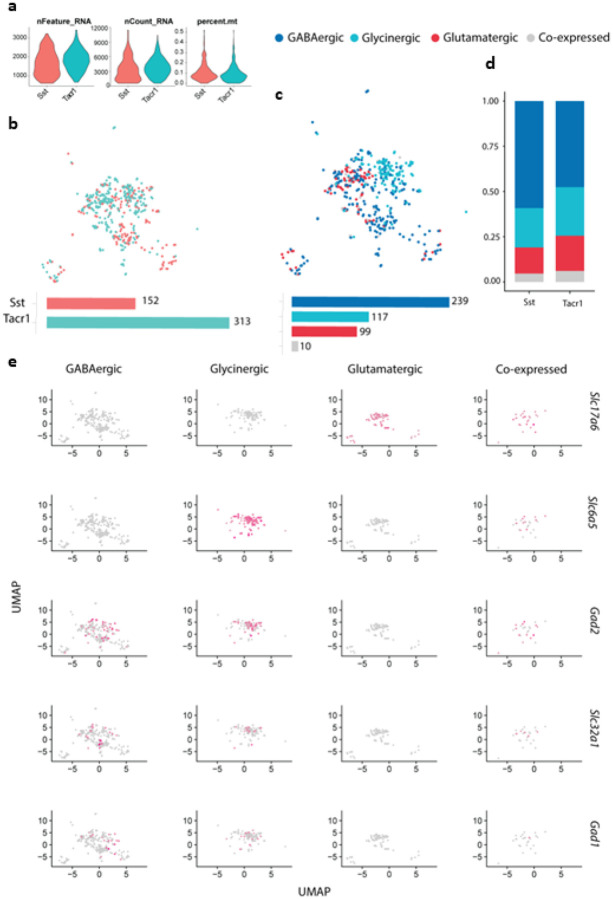
Diversity of Sst^+^ and Tacr1^+^ preBötC/VRC neurons. (a) Violin plots showing quality control metrics for Sst^+^ and Tacr1^+^ populations, including the distributions of detected genes (nFeature RNA), total UMI counts (nCount_RNA), and mitochondrial transcript percentage (percent.mt). (b) UMAP projection of Sst^+^ (salmon) and Tacr1^+^ (teal), with bar chart indicating number of nuclei per population. (c) UMAP projection of neurotransmitter identity showing GABAergic markers *Gad1*, *Gad2*, and *Slc32a1* mixed GABAergic/glycinergic markers in dark blue, glycinergic marker *Slc6a5* in teal, glutamatergic, *Slc17a6* in red), and co-expressed transmitter identity in gray. Bar chart indicates the number of nuclei per neurotransmitter identity. (d) Stacked bar plots showing the proportion of neurotransmitter composition in Sst^+^ and Tacr1^+^ populations. € UMAP feature plots showing expression of canonical neurotransmitter marker genes across the dataset: *Slc17a6*(glutamatergic), *Slc6a5* (glycinergic), *Gad2* and *Gad1*(GABAergic), and *Slc32a1* (vesicular inhibitory transporter). Cells expressing each marker are highlighted in magenta.

Dimensionality reduction using UMAP (resolution = 0.8) showed distinct clustering within this dataset (Fig. S1b). To restrict the analysis to preBötC neurons (*Dbx1*-derived), we excluded cluster 5, identified as cholinergic motor neurons of the Nucleus Ambiguus (NA) based on selective *Chat* expression, likely co-dissected during preBötC tissue enrichment [11] (Fig. S1c). We also excluded cells co-expressing *Sst* and *Tacr1* genes (Fig. S1d-f) as co-expressing cells could confound population classification. In total, 465 nuclei were retained for downstream analysis, with a mean of 1,768 detected genes per nucleus (Fig. 1a, b). Based on exclusive marker expression, 152 nuclei were classified as Sst^+^ and 313 as Tacr1^+^ population (Fig. 1b).

To assess neurotransmitter identity, we examined canonical excitatory and inhibitory markers. Projection of genes *Slc17a6* (*vGlut2*), *Slc6a5* (*GlyT2*), *Gad1*, *Gad2*, and *Slc32a1 (vGat)* onto the UMAP embedding revealed that both Sst^+^ and Tacr1^+^ populations comprise glutamatergic (21%), glycinergic (25%), and GABAergic (51%) phenotypes in mice, with a small fraction (2%) co-expressing multiple neurotransmitter markers (Fig. 1c–g). Despite some overlap, clear differences in neurotransmitter composition were observed between the populations. Tacr1^+^ neurons contained higher proportions of glycinergic, glutamatergic cells and a lower proportion of GABAergic cells compared to the Sst^+^ population. All 465 nuclei were retained for subsequent ion channel and receptor analyses.

### Ion channel transcriptomic distinctions between Tacr1 ^+^ and Sst^+^ populations.

**Figure 2:**
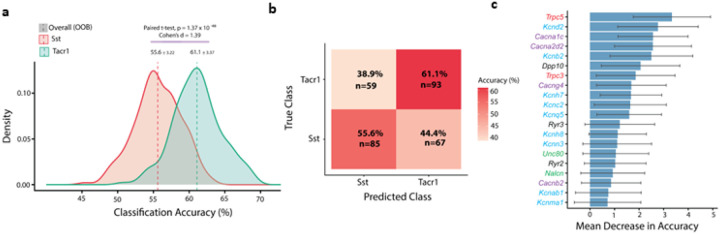
Random Forest classification of Sst^+^ and Tacr1^+^ preBötC/VRC neurons. (a) Distribution of classification accuracy across 200 balanced random forest iterations (n = 152 nuclei per class). Density plots show classification accuracy for Sst^+^ neurons (red), Tacr1^+^ neurons (teal), and overall out-of-bag (OOB) accuracy (gray). Dashed lines indicate mean accuracy for each group. Both populations were classified above chance, with Tacr1^+^ neurons classified with significantly greater accuracy than Sst^+^ neurons across iterations. (b) Confusion matrix summarizing classifier performance across all iterations. Rows indicate true class and columns indicate predicted class. Color intensity reflects classification accuracy (%). Tacr1^+^ neurons were correctly classified at a higher rate (61.1%, n = 93) than Sst^+^ neurons (44.3%, n = 67) indicating an asymmetry in classification performance. (c) Top ion channel genes contributing to classification performance, ranked by mean decrease in accuracy across iterations. Bars indicate the relative importance of each gene in the random forest model, with error bars representing variability across iterations. Colors indicate ion channel family/ functional class. Red- TRP channels; Cyan- various potassium channels; Purple- calcium channels; Black/grey- others; Green-NALCN channelosome

Ion channels are fundamental determinants of intrinsic neuronal excitability, shaping firing patterns and responses to neuromodulation. Given the known functional differences between Sst^+^ and Tacr1^+^ neurons in respiratory rhythm generation, we asked whether distinct ion channel expression profiles could define population specific excitability. To address this, we first assessed whether neurons could be classified based solely on ion channel transcriptomic profiles using a Random Forest (RF) classifier [40]. Across 200 balanced iterations (n = 152 nuclei per group), both populations were classified above chance. Tacr1^+^ neurons were identified with a mean out-of-bag (OOB) accuracy of 61.1% (SD = 3.37%), whereas Sst^+^ neurons showed lower accuracy (55.6% ± 3.32%), yielding an overall mean accuracy of 58.4% (SD = 2.64%; Fig. 2a). Modest accuracy and substantial overlap between populations indicates that ion channel expression differences are graded rather than categorical.

Despite this overlap, we found that classification performance was significantly asymmetric (Δ = 5.49%, Cohen’s d = 1.39, p < 0.001; Fig. 2a), indicating that Tacr1^+^ neurons are more readily distinguishable in ion channel space and this asymmetry was highly consistent across iterations. Receiver Operating Characteristic (ROC) analysis [44, 45] confirmed above-chance discriminability across all iterations, with a mean AUC of 0.615 ± 0.015 (Fig. S2a). The low variance across iterations (Tacr1^+^ SD = 3.37%, Sst^+^ SD = 3.22%) further indicates that this asymmetry is a stable population-level feature rather than a sampling artifact. Consistent with this, Confusion Matrix Analysis (Fig. 2b) showed that Sst^+^ neurons were more frequently misclassified as Tacr1^+^ (44.4%) than the reverse (38.9%), reinforcing the relative distinctness of the Tacr1^+^ population. To confirm that OOB accuracy estimates were not dependent on randomly selected subsets of 152 Tacr1^+^ cells, a stratified held-out evaluation was performed in each iteration. The remaining 161 Tacr1^+^ cells, withheld from training in each iteration, were classified using out-of-sample prediction. This yielded a held-out accuracy distribution that closely overlapped with the OOB estimate (Fig. S2b), confirming that classification performance generalizes to unseen Tacr1^+^ cells and not a sampling artifact of the balanced subset. Top key genes contributing to classification included *Trpc5*, *Kcnd2*, *Cacna2d2*, *Cacna1c*, and *Kcnb2* (Fig. 2c).

**Figure 3:**
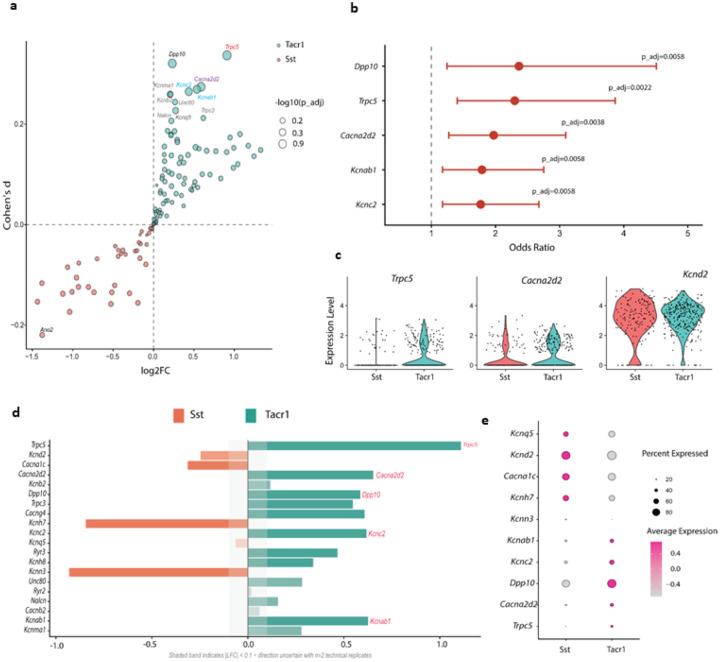
Tacr1^+^ neuronal population are enriched for specific ion channel transcripts in preBötC/VRC. (a) Differential ion channel gene expression between Tacr1^+^ and Sst^+^ neurons. Each point represents an ion channel gene plotted by log_2_ fold change vs effect size (Cohen’s d). Positive values indicate higher expression in Tacr1^+^ neurons, whereas negative values indicate higher expression in Sst^+^ neurons. Point size reflects statistical significance (-log10 adjusted p value). Labeled transcripts represent the enriched candidates. (b) Forest plot showing enrichment of ion channel genes in Tacr1^+^ neurons based on detection rates using Fisher’s exact test (Benjamini–Hochberg corrected). Points represent odds ratios for gene detection in Tacr1^+^ relative to Sst^+^ neurons, with horizontal bars indicating 95% confidence intervals. Dashed vertical line marks OR = 1. (c) Violin plots show normalized expression levels of *Trpc5*, *Cacna2d2*, and *Kcnc2*across nuclei from Sst^+^ and Tacr1^+^ neuronal populations. (d) Pseudobulk log_2_ fold change (LFC) of the top 20 Random Forest discriminatory ion channel genes across two technical replicates following CPM normalization. Bars extending right (teal) indicate higher expression in Tacr1^+^ neurons; bars extending left (coral) indicate higher expression in Sst^+^ neurons. Fisher’s exact significant genes are highlighted in pink italic labels. The shaded band indicates LFC < 0.1, where direction is uncertain given n = 2 technical replicates. (e) Dot plot showing average expression and detection frequency of ion channel genes across Sst^+^ and Tacr1^+^ populations. Dot size reflects the percentage of nuclei expressing each gene and dot color indicates average expression level. Genes are ordered to contrast the enriched (*Trpc5*, *Cacna2d2*, *Dpp10*, *Kcnc2*, *Kcnab1*) and depleted (*Kcnq5*, *Kcnd2*, *Cacna1c*, *Kcnh7, Kcnn3*) ion channel sets in Tacr1_+_ neurons.

We next performed differential expression (DE) analysis focusing on ion channel genes (see Methods). Although individual gene-level effect sizes were modest (log_2_FC = 0.44–0.91; Cohen’s d = 0.27–0.34), a coordinated enrichment pattern emerged in Tacr1^+^ neurons (Fig. 3a and Fig. S3). Enriched genes included potassium channel subunits (*Kcnc2*, *Kcnab1*), the voltage-gated calcium channel auxiliary subunit *Cacna2d2*, and the TRP channel *Trpc5*. While not all genes passed Benjamini–Hochberg correction at padj < 0.05 (Fig. S3), population-level enrichment was supported by Fisher’s exact test with correction. Notably, *Trpc5* showed the strongest enrichment (OR = 2.29, CI [1.40, 3.87], padj = 0.0022), followed by *Dpp10* (OR = 2.37, CI [1.24, 4.51], padj = 0.0058), *Cacna2d2* (OR = 1.97, CI [1.27, 3.10], padj = 0.0038), *Kcnc2* (OR = 1.77, CI [1.17, 2.68], padj = 0.0058), and *Kcnab1* (OR = 1.78, CI [1.17, 2.68], padj = 0.0058; Fig. 3b). These results indicate that enrichment reflects both increased detection frequency and elevated/reduced expression levels in Tacr1^+^ neurons (Fig. 3c).

As an exploratory consistency check, pseudobulk log2 fold changes were computed across two technical replicates following CPM normalization. The analysis confirmed directional consistency with Fisher’s exact results. All five significantly enriched ion channel genes (*Trpc5, Cacna2d2, Kcnc2, Dpp10, Kcnab1*) showed higher expression in Tacr1^+^ neurons across both technical replicates (Fig. 3d). The analysis further revealed the directional contribution of genes selected by the Random Forest classifier. Of the top 20 ion channel genes by RF importance, 12 showed higher expression in Tacr1^+^ neurons and four genes showed higher expression in Sst^+^ neurons (Fig. 3d) indicating that Random Forest classification of Tacr1^+^ neurons is driven by both enrichment of ion channel genes and relative depletion of a subset of channels compared to Sst^+^ neurons (Fig. 3c).

**Figure 4:**
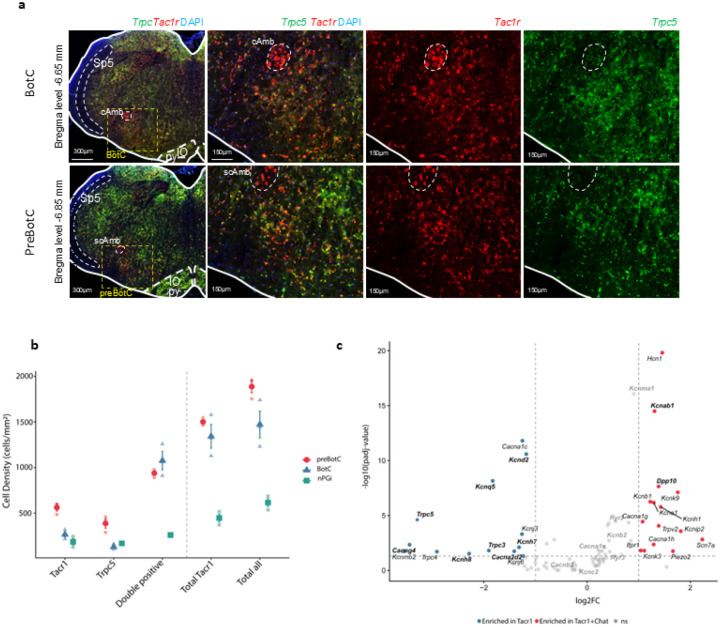
Tacr1^+^ neurons exhibit regional co-expression of *Trpc5* across the ventral respiratory column. (a) Representative coronal sections showing fluorescence *in situ* hybridization (FISH) for *Tacr1* (red) and *Trpc5* (green) with DAPI (blue). Low magnification images (left) outline anatomical boundaries, with dashed lines indicating regions of interest and boxed areas corresponding to higher-magnification views. High magnification merged images (middle) reveal co-localization of *Trpc5* transcripts within Tacr1^+^ neurons. Single-channel images (right) show *Tacr1* and *Trpc5* genes signals separately. Dashed outlines highlight subregions analyzed, including the preBötzinger complex (preBötC). Scale bars as indicated. (b) Cell density (cells/mm^2^) across three brainstem regions: preBötC (red circles), BötC (blue triangles), and nPGi (teal squares). The left panel shows mutually exclusive subpopulations: neurons expressing Tacr1- *Tacr1*^+^*/Trpc5*^−^, Trpc5- *Tacr1*^−^*/Trpc5*^+^, and double-positive neurons co-expressing both markers-Tacr1^+^/Trpc5^+^. The right panel (separated by dashed line) shows aggregate densities: Total Tacr1 (double-positive and Tacr1) and Total all (Tacr1, Trpc5 and Double positive). All values represent cell density normalized to anatomical section area. Individual animal data points are shown with group means ± SEM. (c) Volcano plot showing ion channel differentially expressed genes (DEGs) between cluster 5, identified as a putative Nucleus Ambiguus motor neuron contaminant based on *Chat* gene and the remaining Tacr1^+^ preBötC population. Dashed vertical lines indicate log2FC thresholds of ±1. Dashed horizontal line indicates the significance threshold (padj < 0.05). Genes enriched in the Tacr1^+^ preBötC population are shown in teal; genes enriched in Tacr1^+^+Chat^+^) populations are shown in red, ns-nonsignificant in gray. Note the absence of *Trpc5*^+^ cells in NA (Fig. 4a), consistent with this DEG analysis. Bold gene labels indicate Fisher’s exact test significant genes and RF top features from Fig. 2 and Fig. 3.

To validate these findings, we performed *in situ* hybridization on the top classifier *Trpc5* gene to examine *Trpc5* and *Tacr1* gene co-expression in adult mice and investigated BötC (Bötzinger Complex), preBötC (preBötzinger Complex), and nPGi (nucleus Paragigantocellularis) nuclei (Fig. 4a, b). In the BötC, 179.7 ± 12.1 neurons were double-positive (Tacr1^+^/Trpc5^+^), compared to 44.3 ± 3.8 Tacr1^+^/Trpc5^−^ and 21.7 ± 2.3 Trpc5^+^/Tacr1^−^ neurons. In the preBötC, 136.3 ± 6.4 neurons co-expressed both markers, with 81.3 ± 4.4 Tacr1^+^/Trpc5^−^ and 56.0 ± 6.9 Trpc5^+^/Tacr1^−^ neurons. In the nPGi, 87.3 ± 4.4 neurons were double-positive, with 60.7 ± 6.4 Tacr1^+^/Trpc5^−^ and 56.7 ± 4.1 Trpc5^+^/Tacr1^−^ neurons. Quantification of cell density normalized to anatomical area revealed that double-positive neuron density was markedly higher in the BötC (1077 ± 101 cells/mm^2^) and preBötC (939 ± 26 cells/mm^2^) compared to the nPGi (261 ± 8 cells/mm^2^), providing anatomical validation of the transcriptomic enrichment observed by snRNA-seq (Fig. 4b).

#### Ion channel transcriptomes distinguish Tacr1^+^ preBötC interneurons from Chat^+^/Tacr1^+^ motor neurons

As Random Forest classification identified Tacr1^+^ neurons as transcriptionally homogenous, we next asked whether this reflects intrinsic specialization of preBötC interneurons or a feature shared across Tacr1^+^ expressing populations. We therefore compared ion channel transcriptomes of Tacr1^+^ preBötC interneurons within the double positive Chat^+^-Tacr1^+^ subpopulation (cluster 5; n = 85) (Fig. S1c), which likely corresponding to cholinergic motor neurons of the Nucleus Ambiguus. DE analysis revealed clear divergence, with eight ion channel genes contributing to Tacr1^+^ preBötC classification significantly depleted in the Chat^+^-Tacr1^+^ population. Consistent with this, *in situ* hybridization showed no co-localization of *Trpc5* and *Tacr1* genes in the Nucleus Ambiguus (Fig. 4a), confirming absence of *Trpc5* gene in Tacr1^+^ motor neurons Together, these findings indicate that the ion channel signature underlying Tacr1^+^ classification is circuit-specific and selectively enriched in rhythmogenic preBötC interneurons. This suggests that Dbx1-derived Tacr1^+^ neurons acquire a molecularly distinct ion channel identity not shared by cholinergic Tacr1^+^ neurons outside the rhythmogenic circuit.

#### Enhanced NALCN channelosome subunits assembly in Tacr1^+^ neurons

Given the essential role of NALCN-mediated sodium leak currents in respiratory rhythm generation [28, 31, 46–49], we next examined whether Sst^+^ and Tacr1^+^ neurons differ in expression of all subunits required for assembly of the functional NALCN channel complex, which is required for functional channel assembly [47, 48, 50]. The NALCN channelosome consists of the pore-forming subunit encoded by *Nalcn*, the core auxiliary subunit *Fam155a*, and the auxiliary subunits *Unc79* and *Unc80*, all of which are required for functional channel expression [48, 49].

**Figure 5:**
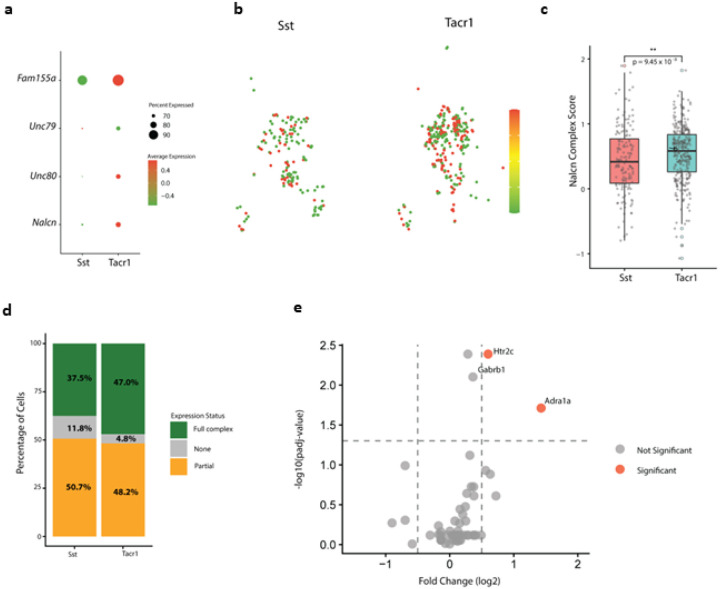
NALCN channelosome expression and neuromodulatory receptor enrichment in Sst^+^ and Tacr1^+^ preBötC/VRC neurons. (a) Dot plot showing the percentage of nuclei and the magnitude of expression difference for genes associated with the *NALCN* channelosome genes *Nalcn, Unc79, Unc80, Fam155a* across populations. The saturation of color represents the average normalized expression level (scaled and centered). (b) UMAP projections showing expression patterns of NALCN channelosome module score across nuclei. For visualization, expression values below the 5th percentile and above the 95th percentile were clipped to reduce the influence of outliers on the color scale. (c) Boxplots show the distribution of *NALCN* channelosome module scores across nuclei. Tacr1^+^ neurons exhibit significantly higher module scores compared to Sst^+^ neurons (Wilcoxon rank-sum test, p = 9.45 × 10^−3^). (d) Proportion of cells expressing the full NALCN channelosome (*Nalcn, Unc79, Unc80, Fam155a*), partial components (missing one or the others), or none of the complex genes in Sst^+^ and Tacr1^+^ populations. Tacr1^+^ neurons show a higher proportion of cells expressing the full set of genes encoding the functional NALCN channelosome. (e) Volcano plot showing expression of neuromodulatory GPCR transcripts, comparing Tacr1^+^ versus Sst^+^ neurons. Dashed lines indicate thresholds for significance and fold change.

To quantify coordinated expression of the NALCN channelosome, we derived single-cell module scores using “AddModuleScore” *(Nalcn, Unc80, Unc79, and Fam155a)* as implemented in Seurat. Tacr1^+^ neurons exhibited significantly higher module scores than Sst^+^ neurons (Wilcoxon rank-sum test, p = 9.45 × 10^−3^; Fig. 5a–c). Consistent with this, 47.0% of Tacr1^+^ neurons expressed the full NALCN channelosome genes compared to 37.5% of Sst^+^ neurons, whereas partial complex expression was similar between groups (Tacr1^+^: 48.2%; Sst^+^: 50.7%) (Fig. 4d). Cells lacking detectable expression of any NALCN-related subunits were rare in both populations but were slightly more frequent among Sst^+^ neurons (11.8% vs. 4.8% in Tacr1^+^ neurons) (Fig. 4d). These results were not confounded by sequencing depth, as NALCN module scores showed no correlation with total UMI counts (r = 0.027, p = 0.57; Fig. S4). Together, these findings indicate that while both populations express components of the NALCN channelosome, a larger fraction of Tacr1^+^ neurons co-express the full complement of genes required for functional channel assembly

Because NALCN activity is strongly regulated by G protein–coupled receptor (GPCR) signaling [46, 51, 52]. We next asked whether Sst^+^ and Tacr1^+^ neurons differ in neuromodulatory receptor expression. DE analysis identified selective enrichment of *Htr2c* (5-HT2C receptor; padj = 0.004) and *Adra1a* (α1A adrenergic receptor; padj = 0.019) in Tacr1^+^ neurons (Fig. 5f), suggesting enhanced serotonergic and noradrenergic modulation in this population. Together, these data reveal distinct molecular specializations between Tacr1^+^ and Sst^+^ neurons in the preBötC, providing testable hypotheses for differential neuromodulatory control of respiratory rhythm-generating circuits.

## Discussion

Over the past two decades, Sst^+^ and Tacr1^+^ neuronal populations in the ventrolateral medulla have been central to canonical models of respiratory rhythm generation and modulation [2, 11, 12, 14, 15, 17, 30, 53–55]. Lesion and genetic fate-mapping studies have demonstrated that normal rhythmic breathing critically depends on these genetically defined neuronal populations localized within the preBötC [11, 12, 15, 17, 18, 22, 30, 31, 56]. Building on this foundation, our single-nucleus transcriptomic profiling of Sst^+^ and Tacr1^+^ neuronal populations in the preBötC of the mouse medulla provides a molecular framework for this rhythmogenic circuitry. The data reveals a population-specific repertoire of ion channels and neuromodulatory receptors, providing insight into how these historically defined populations differentially contribute to respiratory control.

Across both differential expression (DE) and Random Forest (RF) classification, Tacr1^+^ and Sst^+^ neuronal populations occupy distinct molecular states within the respiratory network, distinguished by their ion channel repertoires. The greater ability to classify Tacr1^+^ neurons relative to Sst^+^ neurons using RF reflects a greater homogeneous ion channel transcriptomic identity. This supports reliable modulation of network dynamics, including the emergence of the percolation phase that precedes the generation of the inspiratory burst (Fig. 2) [5, 25, 27, 57, 58]. Notably, this identity was consistent across 200 iterations, as indicated by the low variance of RF importance scores, supporting a stable population-level feature rather than a sampling artifact (Fig. 2). Among the top discriminatory features, Tacr1^+^ neurons showed enrichment of ion channels and auxiliary subunits associated with enhanced excitability and high-frequency firing capacity, including *Trpc5, Kcnc2*, and *Cacna2d2* (Fig. 2c). To validate these findings, XGBoost (gradient-boosted decision tree ensemble) classification was performed using identical balanced subsampling parameters. It identified the same top discriminatory ion channel genes, supporting the robustness of the molecular signature across independent classification frameworks (Fig. S5).

Pseudobulk log2 fold change analysis further revealed that *Kcnd2* (Kv4.2), *Cacna1c* (L-type Cav1.2), *Kcnh7* (ERG-family), and *Kcnn3* (SK3) were expressed at higher levels in Sst^+^ neurons, indicating that their contribution to RF classification algorithms reflects depletion relative to Tacr1^+^ neurons rather than enrichment. These features are consistent with a reduced capacity for spike frequency adaptation and afterhyperpolarization in Tacr1^+^ neurons (Fig. 3d,e) [59]. By contrast, the lower classification accuracy suggests greater ion channel heterogeneity in Sst^+^ neurons indicating a more diverse and flexible functional role within the preBötC network (Fig. 2a,b). This is consistent with findings from de Sousa Abreu et al. (2022), who demonstrated that preBötC Sst^+^ neuron activation produces probabilistic, state-dependent effects on breathing pattern. Together, these findings suggest that Sst^+^ neurons constitute a functionally heterogeneous ensemble whose diverse ion channel transcriptomes underlie a flexible and state-dependent modulatory role in the preBötC [4].

Our results from DE and RF classification identifies a consistent set of ion channel genes enriched in Tacr1^+^ neurons, providing further support for a molecularly distinguishable in Tacr1^+^ ion channel signature (Fig. 2, Fig. 3). Among these features is *Kcnc2*, which encodes the Kv3.2 subunit. Kv3 channels are characterized by high activation thresholds and rapid deactivation kinetics, enabling fast action potential repolarization and short refractory periods [60, 61]. In the context of respiratory rhythm generation, such properties may support high-frequency and temporally precise firing within the preBötC network. We also find enrichment of *Trpc5* in Tacr1^+^ neurons. TRPC5 is a calcium-permeable, Gq/PLC-coupled channel associated with slow depolarizing currents and sustained excitability in central neurons [62–66]. *In vivo* studies have implicated TRPC5 in autonomic regulation, including effects on heart rate and reflexive bradycardia [67–70], suggesting that its enrichment in Tacr1^+^ neurons may contribute to integrative cardiorespiratory modulation. Importantly, the co-enrichment of *Trpc5* and *Tacr1* genes in the same population suggests a receptor–effector coupling mechanism with direct functional implications for intrinsic rhythmogenesis. Substance P (SP), acting through the NK1/Tacr1 receptor, signals via Gq/PLCβ, and may recruit and modulate TRPC5 (receptor operated cation channels), producing a sustained inward cation current that depolarizes the membrane and support repetitive bursting [62, 64]. This pathway is consistent with the calcium-activated non-selective cation current (I_CAN_) first described in preBötC pacemaker neurons under synaptic blockade by Peña and Ramirez (2004). The potential contribution of the I_CAN_ in respiratory rhythm generation, its role in the generation of the inspiratory burst, as well as the molecular identity of I_CAN_ have been studied extensively, yet many questions remain [71–75]. Our transcriptomic data raises the possibility that TRPC5 constitutes at least part of the molecular substrate for I_CAN_ in Tacr1^+^ neurons. Neuronal preBötC population with these properties may contribute to the SP-induced intrinsic bursting observed under TTX conditions. Although beyond the scope of the present study, this hypothesis could be tested with pharmacological blockers for TRPC5 [76, 77] during SP modulation under conditions of synaptic isolation [28].

We further identified the presence of *Cacna2d2*, encoding the α2δ auxiliary subunit of P/Q-type, N-type and L-type voltage-gated calcium channels, which may modulate trafficking and surface expression of Cav channels, potentially influencing activity-dependent Ca^2+^ influx and synaptic transmission within Tacr1^+^ neurons. Collectively, these findings suggest that Tacr1^+^ neurons are molecularly specialized for a variety of mechanisms that could dynamically modulate respiratory output [25, 27].

Mechanistically, TACR1 signaling is known to modulate NALCN-mediated sodium leak currents, generating a persistent inward current that depolarizes resting membrane potential and promotes inspiratory bursting [31, 56]. NALCN is a voltage-independent cation channel highly expressed in the preBötC (Yen, et al., 2017). Knockout studies show conditional deletion of NALCN results in recurring apneas and neonatal death within 24 hours after birth (Lu et al., 2009). Consistent with this, enrichment of genes encoding complete NALCN channelosome in Tacr1^+^ neurons supports a mechanism by which SP can reinforce tonic depolarizing drive by modulation of NALCN during Tacr1^+^ activation (Fig. 5a–d) [23, 28, 31, 46]. Taken together with the *Trpc5*-gene enrichment described above, our data suggest that SP may engage in at least two parallel depolarizing mechanisms in Tacr1^+^ neurons: tonic NALCN-mediated sodium leak and receptor-operated TRPC5-mediated cation influx. These mechanisms are not mutually exclusive and may act synergistically to lower burst threshold and sustain rhythmic activity across different neuromodulatory states. We also found significant expressions of transcripts encoding the *Htr2c* serotonin and alpha-1A adrenergic receptors (Fig. 5f), suggesting additional potential mechanisms for modulating *NALCN* activity in shaping the excitability of Tacr1^+^ neurons. Overall, our study defines genes that can regulate the excitability of Tacr1^+^ neurons and reveal a molecular signature, enriched in K^+^ channels, TRP channels, and neuromodulatory receptors.

Finally, we would like to acknowledge various limitations and caveats. First, our dataset is derived from neonatal mice (P5–P10), which will likely not fully capture the properties of mature circuit states. However, the shared Dbx1 lineage of Sst^+^ and Tacr1^+^ populations make systematic developmental bias unlikely, and the convergence of results across multiple analytical approaches supports a cell-type–specific interpretation. Second, single-nucleus RNA sequencing is subject to dropout (transcriptional absence cannot be differentiated from true absence), which may obscure low-abundance transcripts. Nevertheless, the consistent identification of key ion channel genes across 200 independent subsampling iterations, together with concordant differential expression and enrichment analyses, argues against dropout-driven artifacts.

Notably, ion channel differential expression analysis of cluster 5, identified as a putative Nucleus Ambiguus motor neuron contaminant, revealed depletion of key Tacr1^+^ preBötC markers including *Trpc5* and *Cacna2d2* genes and enrichment of distinct ion channel transcripts including *Hcn1* and *Scn7a*, further supporting the molecular specificity of the preBötC Tacr1^+^ ion channel signature (Fig. 4c). In summary, our study establishes a molecular framework distinguishing Tacr1^+^ from Sst^+^ neurons in the preBötC. Despite their shared developmental origin, Tacr1^+^ neurons exhibit a distinct ion channel and neuromodulatory receptor signature that likely underlies its specialized contribution to respiratory rhythm generation and modulation.

## Supplementary Material

Supplementary Files

This is a list of supplementary files associated with this preprint. Click to download.

- Fig.S1.pptx
- Fig.S3.pptx
- Fig.S4.pptx
- TableS1.csv
- Fig.S2.pptx
- Fig.S5.pptx

## Data Availability

The datasets used and analyzed during the current study are available from the corresponding author upon reasonable request, as the data are shared across multiple ongoing projects in the lab.
